# Diverse Bacteria with Lignin Degrading Potentials Isolated from Two Ranks of Coal

**DOI:** 10.3389/fmicb.2016.01428

**Published:** 2016-09-09

**Authors:** Lu Wang, Yong Nie, Yue-Qin Tang, Xin-Min Song, Kun Cao, Li-Zhu Sun, Zhi-Jian Wang, Xiao-Lei Wu

**Affiliations:** ^1^School of Earth and Space Sciences, Peking UniversityBeijing, China; ^2^College of Engineering, Peking UniversityBeijing, China; ^3^College of Architecture and Environment, Sichuan UniversityChengdu, China; ^4^State Key Laboratory of Enhanced Oil Recovery, Research Institute of Petroleum Exploration and DevelopmentBeijing, China; ^5^Xinchun Production Plant, Sinopec Shengli Oilfield, KaramayChina

**Keywords:** lignin degradation, coal, laccase, LMCO gene, seed bank

## Abstract

Taking natural coal as a “seed bank” of bacterial strains able to degrade lignin that is with molecular structure similar to coal components, we isolated 393 and 483 bacterial strains from a meager lean coal sample from Hancheng coalbed and a brown coal sample from Bayannaoer coalbed, respectively, by using different media. Statistical analysis showed that isolates were significantly more site-specific than medium-specific. Of the 876 strains belonging to 27 genera in Actinobacteria, Firmicutes, and Proteobacteria, 612 were positive for lignin degradation function, including 218 strains belonging to 35 species in Hancheng and 394 strains belonging to 19 species in Zhongqi. Among them, the dominant lignin-degrading strains were *Thauera* (Hancheng), *Arthrobacter* (Zhongqi) and *Rhizobium* (both). The genes encoding the laccases- or laccase-like multicopper oxidases, key enzymes in lignin production and degradation, were detected in three genera including *Massila* for the first time, which was in high expression by real time PCR (qRT-PCR) detection, confirming coal as a good seed bank.

## Introduction

Coal is a mixture of many organic compounds, including those with molecular structures similar to lignin, which is a complex aromatic heteropolymer composed of phenylpropanoid aryl-C_3_ units linked by a variety of ether and carbon-carbon bonds (Bugg et al., 2011). These lignin-like complex polymers are highly resistant to breakdown, protecting coal from being degraded. In the last decades, microbial degradation of lignin has been intensively studied in white-rot and brown-rot fungi (Madhavi and Lele, 2009; Kudanga et al., 2011). In fungi, a range of extracellular lignolytic enzymes have been reported (Sanchez, 2009), including lignin peroxidases, Mn peroxidases, and laccases- or laccase-like multicopper oxidases (LMCO), which are involved in the process of depolymerization and lignin degradation (Sanchez, 2009). Recently, a large list of bacteria able to break down lignin was reported (Bugg et al., 2011), including *Streptomyces viridosporus* T7A, *Nocardia* *autotrophica*, *Sphingobium* sp. SYK-6, *Pseudomonas putida* mt-2, *Rhodococcus* sp., *Burkholderia cepacia*, *Microbacterium* sp., and *Citrobacter* sp. The enzymes for lignin degradation in bacteria are poor understood, limited amount of paper reported that bacteria might use the extracellular lignin-degrading enzymes similar to fungi (Bugg et al., 2011). In addition, almost all bacterial strains displaying laccase activity possess at least one laccases- or laccase-LMCO gene (Kellner et al., 2008; Fang et al., 2011; Kudanga et al., 2011). However, only a few bacteria encoding laccase enzymes have been reported, including *Azospirillum lipoferum, Marinomonas mediterranea, S. griseus, S. lavendulae, Ralstonia solanacearum, Sinorhizobium meliloti*, and *Bacillus subtilis* (Sharma et al., 2007; Kellner et al., 2008; Madhavi and Lele, 2009; Bugg et al., 2011). A search of protein data has suggested that laccases are present in other bacterial species, such as *B. subtilis*, *Escherichia coli, Mycobacterium tuberculosum, P. syringae*, and *P. aeruginosa* (Gennaro et al., 2011). It is noticed that these bacterial strains with lignin degradation abilities were mainly isolated from the guts of termites and wood-boring beetles, and belonged to three classes, Actinomycetes, Alphaproteobacteria, and Gammaproteobacteria (Bugg et al., 2011).

Since the molecular structure of coal components are similar to that of lignin, natural coal could be used a “seed bank” of bacterial strains (Cai et al., 2015) able to degrade lignin. In fact, coal microbial communities have been reported from different sites in the world (Shimizu et al., 2007; Penner et al., 2010; Beckmann et al., 2011; Tang et al., 2012; Stepniewska et al., 2014), which reported the lignin degradation potentials in the coal microbial community. Hence, we took two ranks of coal, a meager lean coal from Hancheng coalbed and a brown coal from Bayannaoer coalbed (Tang et al., 2012) as seed banks to isolate bacterial strains with lignin degrading potentials. The lignin degradation abilities of the isolated strains as well as the LMCO genes were detected in order to understand the lignin degrading potentials of the isolates. From the two coal samples, we isolated 876 strains by using three kinds of media, coal medium (M), mineral medium (W), and coal/mineral medium (MW). Among the 876 strains, 612 strains were positive for lignin degradation to varying degrees, confirming coal as a good seed bank for lignin-degrading bacteria.

## Materials and Methods

### Coal Samples

Two coal samples were collected from coalbeds in Hancheng (110°45′E, 35°47′N), Shanxi Province, and Bayannaoer (108°11′E, 40°45′N) in the Inner Mongolia Autonomous Region, located at the southeastern and northwestern borders of the Ordos Basin, respectively. The Hancheng coal (H) was a meager lean coal formed in the Carboniferous–Permian period from marine-continental interactive sedimentation. The sample was collected from a coal seam depth of 651–652 m with an *in situ* temperature of 26.7°C. The Bayannaoer coal (Zhongqi, ZQ) was brown coal formed in the middle and lower Jurassic period from continental sedimentation. The sample was collected from a 38–40 m deep coal seam with an *in situ* temperature of 20.5°C. Both coals were sampled as big intact blocks (cubes of approximately 10 cm × 10 cm × 10 cm), directly from coal seams which were in production. Coal blocks were immediately put into sterile plastic bags and taken to the lab at 5–7°C within 24 h. Approximately 2 cm of the outer layer of the samples was removed with sterile tools, as the inner part was stored aseptically at -80°C until culture. The coal properties, including moisture, ash content, volatile matter content, sulfur content, and gas content were detected as reported by Tang et al. (2012). The mixed interlayer water was collected from different layers in Hancheng area for media preparation.

### Strain Isolation and Identification

About 60 g of each coal sample was grinded using sterile mortars and screened with 16 meshes. The coal powder was then mixed with 300 ml of interlayer water. The mixer was rotated at a speed of 800 rpm at 30°C for 20 h. After natural precipitation, 1 ml of supernatant was removed and serially diluted. Three kinds of media were used to isolate bacteria from all samples: (i) coal medium (M) (60 g coal with 300 ml interlayer water mixed and rotated for 20 h in a 30°C water bath, and then sterilized at 121°C for 1 h; the mixture was centrifuged and the supernatant was diluted 10-fold; agar was added at 2.0% w/v, and media was again sterilized); (ii) mineral medium (W) [L^-1^: 5 g NaCl, 1 g K_2_HPO_4_, 1 g NH_4_H_2_PO_4_, 1 g (NH_4_)_2_SO_4_, 0.2 g MgSO_4_⋅7H_2_O, 3 g KNO_3_, and 20 g agar, sterilized at 121°C for 1 h]; and (iii) coal/mineral medium (MW) [5 g NaCl, 1 g K_2_HPO_4_, 1 g NH_4_H_2_PO_4_, 1 g (NH_4_)_2_SO_4_, 0.2 g MgSO_4_⋅7H_2_O, 3 g KNO_3_, and 20 g agar in 1 L M medium and sterilized at 121°C for 1 h]. Each sample was serially diluted and spread on the three media agar plates in triplicate, and cultured at 30°C for 3–8 days. All isolates growing on the plates were picked and transferred to the same freshly prepared media, until pure cultures were obtained; isolates were stored for further investigation. The isolates were named after the sample location, medium name, and the colony series numbers. For example, the first and fifth strains originating from Hancheng, and isolated from M medium, were named as HM1 and HM5, respectively.

### DNA Extraction and PCR Amplification of 16S rRNA Gene

A single colony was transferred to a glass tube with 1 ml of liquid medium (corresponding to the original medium used for isolation) without agar. The cultures were incubated for 3–5 days at 30°C with shaking at 180 rpm. The cultured cells were collected and concentrated and the extraction of genomic DNA and PCR amplification of the 16S rRNA gene were performed as described by Wang et al. (2007). The 16S rRNA gene was amplified from genomic DNA of all isolates using a bacterial universal primer set as described previously (Wang et al., 2007) (**Supplementary Table S1**). The PCR products were purified with the QIAquick PCR Purification Kit (Qiagen, Shanghai, China) and digested with *Rsa* I and *Msp* I (TaKaRa, Dalian, China). The isolates were digested firstly by *Rsa* I at 37°C for 5 h, then the isolates with same restriction enzyme digestion patterns were selected to be digested by *Msp* I at 37°C for 5 h. The total reaction system was 20 μl with 5 μl PCR products, 2 μl 10 × buffer (including 0.1% BSA), 0.5 μl restriction endonucleases and 12.5 μl deionized water. According to patterns of 16S rRNA gene fragment restriction digestion. The 16S rRNA genes, from 876 isolates, were classified into 77 patterns, and the PCR products of each pattern were further sequenced (Cook and Meyers, 2003; Sun et al., 2013). The obtained DNA sequences were aligned in GenBank using the BLAST tools^1^. The reference sequences were retrieved from GenBank. After multiple sequence alignment of the sequences by CLUSTAL X and manually correction, 1283 bp was selected to construct phylogenetic tree using the neighbor-joining method in the MEGA software package version 5.0 (Tamura et al., 2011).

To rapidly distinguish bacterial species and strains of the dominant *Thauera* genus, we randomly selected 145 of 224 total *Thauera* isolates (according to 16S rRNA gene sequences and 16S rRNA gene fragment restriction digestion patterns), and investigated their distributions of repetitive DNA elements in the 16S rRNA genes according to BOX-PCR and repetitive extragenic palindromic PCR (REP-PCR) patterns. Primers (Sangon, Shanghai, China) used for BOX-PCR and REP-PCR are listed in **Supplementary Table S1**. Amplification was performed using rTaq DNA polymerase (TaKaRa, Dalian, China) in a MJ Mini Personal Thermal Cycler (Bio-Rad, Hercules, CA, USA) as follows: (1) BOX-PCR: 5 min at 95°C, 35 cycles of 1 min at 53°C, 4 min at 72°C, and 4 min at 72°C, with a single final extension of 10 min at 72°C; (2) REP-PCR: 5 min at 94°C, 30 cycles of 30 s at 95°C, 60 s at 45°C, and 8 min at 65°C, with a single final extension 16 min at 65°C. The reaction products were stored at 4°C until they were subjected to gel electrophoresis using a 2.0% (w/v) agarose gel with a 100 bp DNA ladder (Tiangen, Beijing, China).

### Lignin-Degrading Abilities

To detect the ability of bacterial oxidation of lignin coupled to the reduction of molecular dioxygen to water (Couto and Herrera, 2006), GU-WA medium (10 g NaH_2_PO_4_⋅12H_2_O, 2 g KH_2_PO_4_, 0.5 g NaCl, 0.5 g NH_4_NO_3_, 0.5 g MgSO_4_⋅7H_2_O, 0.1 mg FeSO_4_7⋅H_2_O, 0.1 mg CaCl_2_, 1 mg CuSO_4_, 0.2 g guaiacol, 2 g 80 mesh Eucalyptus wood powder, and 20 g agar in 1 L distilled water, adjusted to a final pH of 7.0 and then sterilized; Jung et al., 1995; Li et al., 2006), was prepared for culturing 77 representative strains (based on 77 patterns achieved through restriction digestion of PCR-amplified 16S rRNA gene) from Hancheng and Zhongqi. The strains were pre-cultured in W medium with 0.01% yeast extract and 0.01% tryptone for 3 days, after which 3 μl inoculum was transferred to GU-WA medium and cultured at 30°C. After 7–10 days, the color of the circle surrounding inoculation sites was assessed for the presence or absence of a color change.

### Analysis of LMCO Gene

Previously described primers used to amplify the LMCO gene are listed in **Supplementary Table S1**. DNA fragments corresponding to the correct target size for each gene were cloned into the pGEM-T Easy Vector System I (Promega, Madison, WI, USA) and sequenced. The obtained DNA sequences were aligned in GenBank using the Blastx ^footnotenumfont 1^. The functionally identified reference sequences or those with the highest similarities to the query sequences were retrieved from GenBank. All sequences were aligned using CLUSTAL X with manual correction. The phylogenetic tree of the LMCO genes based on amino acid sequences was constructed using the neighbor-joining method in the MEGA software package version 5.0 (Tamura et al., 2011). Trees were bootstrapped using 1000 replications. The stability of tree topology was evaluated with maximum-likelihood and maximum parsimony algorithms.

### Analysis of LMCO Gene Expression

One strain *Massilia* sp. ZQW3 was inocubated by Luria-Bertani (LB) liquid medium at 30°C for 3 days with shaking of 180 rpm. Bacteria cells were collected (3200 × g, 5 min) and washed three times by basal salt medium (10 g NaH_2_PO_4_⋅12H_2_O, 2 g KH_2_PO_4_, 0.5 g NaCl, 0.5 g NH_4_NO_3_, 0.5 g MgSO_4_⋅7H_2_O, 0.1 mg FeSO_4_⋅7H_2_O, 0.1 mg CaCl_2_, 1 mg CuSO_4_ in 1 L distilled water, adjusted to a final pH of 7.0). Then cells were inocubated to basal salt medium with lignin (10 g/L) and glucose (10 g/L) as sole carbon source, respectively. The final optional density (OD) was 1.0. The cultures were incubated for 24 h at 30°C with shaking at 180 rpm, and the total RNAs were obtained according to RNAprep Pure Cell/Bacteria Kit (TIANGEN). Primers used in quantitative real-time PCR (qRT-PCR) were *16S r*RNA-F (5′-CTCCTACGGGAGGCAGCAGT-3′), *16S r*RNA-R (5′-CGTATTACCGCGGCTGCTGG-3′), *LMCO*-F (5′-ATCCTGGTGCC-GGCGGAGAT-3′), and *LMCO*-R (5′-GTTTTGCTGGAGCTTGAACT-3′).

The reverse transcription PCR for complementary DNA (cDNA) was performed followed by qRT-PCR using the SYBR^®^ Green method as the instruction of SYBR Premix ExTaq^TM^ GC (TaKaRa). Cycling parameters of qRT-PCR reactions were programmed with an initial step of 30 s at 95°C followed by 50 cycles consisting of denaturation at 95°C for 5 s, annealing at 55°C for 30 s, then 10 s at 95°C, melt curve 65 to 95°C, increment 0.5°C for 5 s. The relative quantification was analyzed with the 2^-ΔΔCt^ method. Technical triplicates were performed for each biological replicate, and the average values were used for quantification. *16S rRNA* herein was used as internal control to normalize the relative transcription of the analyzed gene.

#### Sequence Deposited

The GenBank accession numbers for the 16S rRNA gene sequences of the strains isolated in this study are listed in **Supplementary Table S2**. The GenBank accession numbers for the LMCO gene sequences of the strains isolated in this study are listed in **Supplementary Table S3**.

## Results

### The Diversity of Bacterial Isolates from Two Coals in Ordos Basin

**FIGURE 1:**
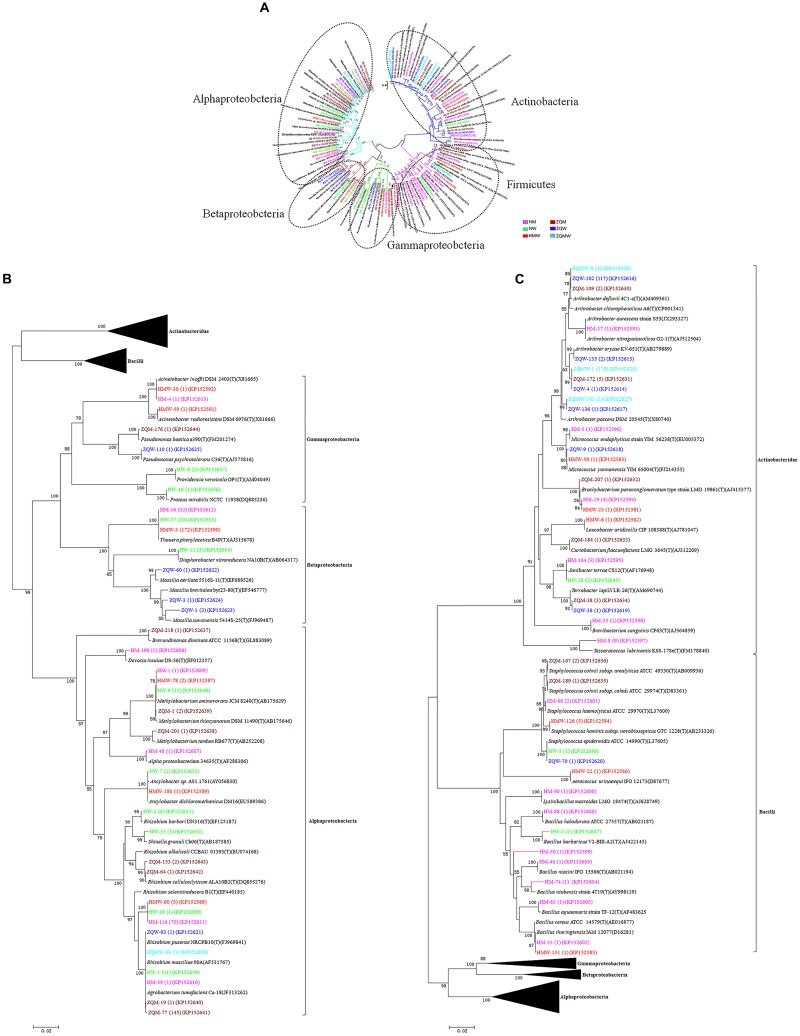
**Phylogenetic tree showing the genetic relationships.** **(A)** The tree about all the isolates. **(B)** The tree about the isolates affiliated with the class Actinobacteridae and Bacilli. **(C)** The tree about the isolates affiliated with the class Alphaproteobacteria, Betaproteobacteria, and Gammaproteobacteria. The trees were constructed by the Neighbor-Joining method using partial sequences of 16S rRNA gene. Bootstrap probabilities >70% are indicated at the branch nodes. Numbers of isolates with identical sequences, the GenBank accession numbers for reference strains and isolates obtained in this study are shown in parentheses.

**FIGURE 2:**
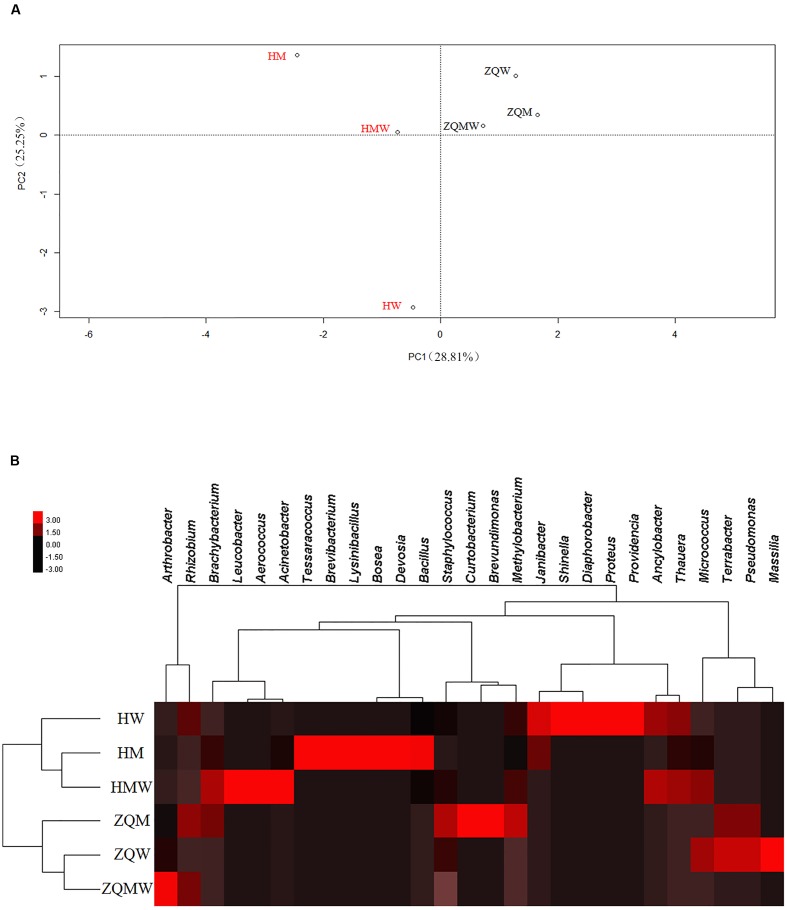
**Statistical analyses of isolates from different samples in different media.** **(A)** Principal component analysis (PCA) of isolates from all the media. **(B)** Heat map of all the media, which was generated in CLUSTER and visualized using TREEVIEW 1.1.5 R2.

A total of 393 isolates and 483 isolates were obtained from the coal of Hancheng and Zhongqi, respectively. The 16S rRNA genes were amplified by PCR and classified according to 16S rRNA gene fragment restriction digestion patterns (using two endonuclease cleaving enzymes). One 16S rRNA gene amplicon of each pattern was selected randomly for sequencing. Through sequencing and alignment, the sequences having >97% similarity were grouped into a cluster. Based on restriction enzyme digestion results and sequencing results, all bacterial strains could be classified into 77 clusters, which contained 27 genera from Actinobacteria (339 isolates from 8 genera), Firmicutes (23 isolates from 5 genera), and Proteobacteria (514 isolates from 14 genera) (**Figures 1A–C**; **Table 1**). These isolates from Hancheng and Zhongqi showed 98.80–100.00% and 98.00–100.00% 16S rRNA gene identities to valid reference strains, respectively. Principal component analysis and clustering analysis were performed on all the bacterial patterns (**Figures 2A,B**), which showed that isolates were site-specific more significantly than medium-specific.

**Table 1:** Classification of bacterial in the coal and number of isolates with diversity for the coal samples studied.

Taxonomy	HM	HW	HMW	ZQM	ZQW	ZQMW
**Actinobacteria**	**19**	**2**	**3**	**12**	**123**	**181**
Actinobacteridae	19	2	3	12	123	181
**Firmicutes**	**9**	**2**	**7**	**3**	**1**	**0**
Bacilli	9	2	7	3	1	0
**Proteobacteria**	**107**	**62**	**182**	**154**	**8**	**1**
Alphaproteobacteria	74	36	8	153	2	1
Betaproteobacteria	32	23	172	0	5	0
Gammaproteobacteria	1	3	2	1	1	0
Number of total isolates	135	66	192	169	132	182
Shannon-Weiner Index	2.467	2.311	2.369	2.061	1.799	0.562


*The bold values of “Actinobacter, Firmicutes, and Proteobacteria” indicating the taxonomy of isolates are in the phylum level.*

**FIGURE 3:**
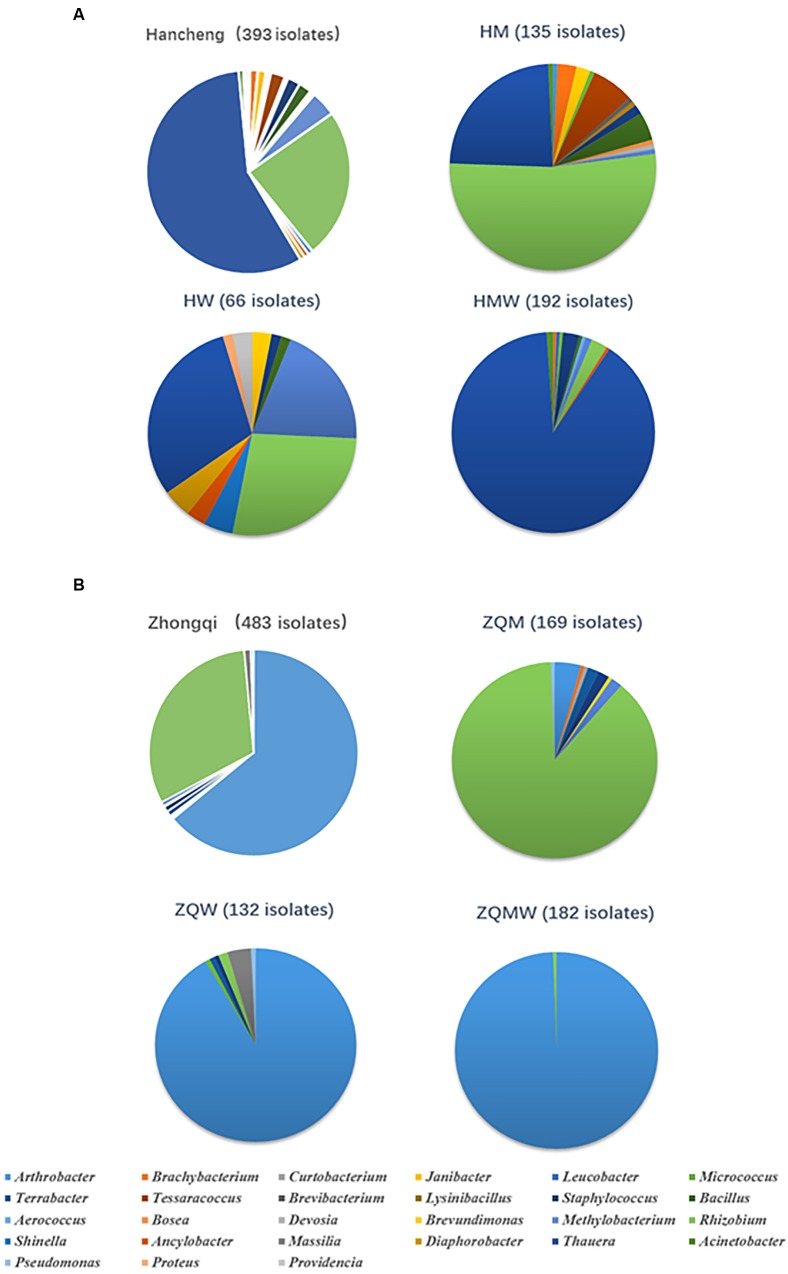
**Abundance of bacteria in Hancheng and Zhongqi samples.** **(A)** Abundance of strains isolated from Hancheng, HM, HW, and HMW. **(B)** Abundance of strains isolated from Zhongqi, ZQM, ZQW, and ZQMW.

**FIGURE 4:**
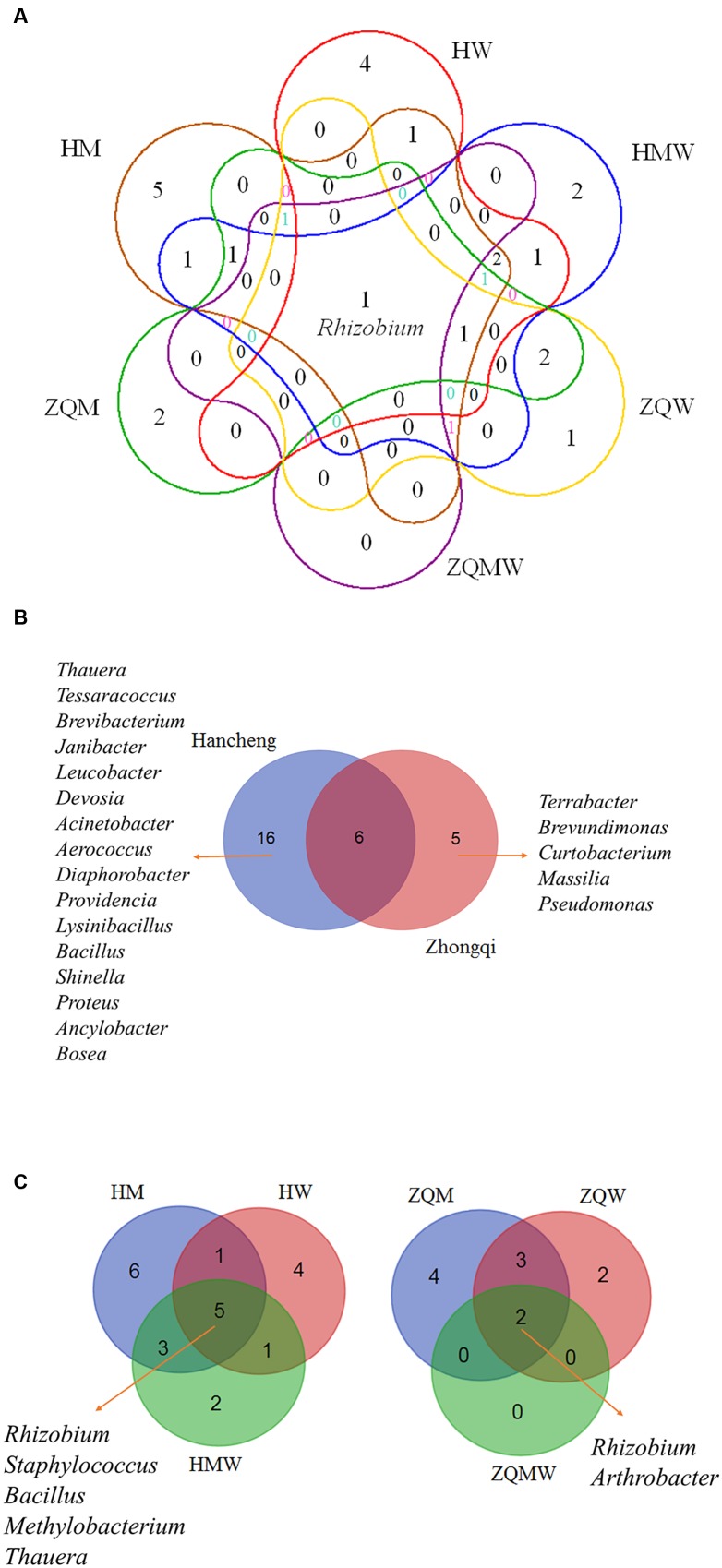
**Venn diagram showing the shared and different bacterial genera.** **(A)** All studied samples in different media, **(B)** Hancheng and Zhongqi and **(C)** HM, HW and HMW, and ZQM, ZQW, and ZQMW.

Isolates from the genus *Thauera* dominated those from Hancheng, accounting for 57% of the total; the remaining isolates were from the genera *Arthrobacter, Rhizobium, Staphylococcus, Brachybacterium, Micrococcus*, *Methylobacterium, Tessaracoccus, Brevibacterium, Janibacter, Leucobacter, Devosia, Acinetobacter, Aerococcus, Diaphorobacter, Providencia, Lysinibacillus, Bacillus, Shinella, Proteus, Ancylobacter*, and *Bosea* (**Figure 3A**). In contrast, *Arthrobacter* dominated all isolates from Zhongqi (64%), and the remaining isolates were *Rhizobium, Staphyloco-ccus, Brachybacterium, Micrococcus*, *Methylobacterium, Terra-bacter, Brevundimonas, Curtobacterium, Massilia*, and *Pseudom-onas* (**Figure 3B**). Isolates from Hancheng coal had higher Shannon-Weiner index values, 2.83, at the 16S rRNA gene similarity level, compared to 2.13 of Zhongqi coal (**Table 1**), suggesting higher bacterial diversity in Hancheng. Strains belonging to *Arthrobacter, Rhizobium, Staphylococcus, Brachybacterium, Micrococcus*, and *Methylobacterium* genera were isolated from both Hancheng and Zhongqi, which occupied 32.06 and 97.31% of total isolates, respectively. Strains belonging to *Rhizobium* could be isolated from all the media from both Hancheng and Zhongqi (**Figure 4A**). *Thauera, Tessaracoccus, Brevibacterium, Janibacter, Leucobacter, Devosia, Acinetobacter, Aerococcus, Diaphorobacter, Providencia, Lysinibacillus, Bacillus, Shinella, Proteus, Ancylobacter*, and *Bosea* were only found in Hancheng (**Figure 4B**; **Supplementary Table S4**), and *Terrabacter*, *Brevundimonas*, *Curtobacterium*, *Massilia*, and *Pseudomonas* were only found in Zhongqi (**Figure 4B**; **Supplementary Table S4**).

### The Influence of Media in the Diversity of Bacterial Isolates

In this study, we used three different types of media to isolate bacteria from coal. There were 135, 66, and 192 isolates obtained from Hancheng using M, W, and MW media, respectively. The Shannon-Weiner index values of the isolates using M, W, and MW media were 2.467, 2.311, and 2.369, respectively (**Table 1**). This showed the highest diversity of isolates was obtained using M medium and the lowest diversity was obtained using W medium. In addition, M medium also obtained the highest diversity of isolates from Zhongqi. The Shannon-Weiner index values of isolates from Zhongqi using M, W, and MW media were 2.061, 1.799, and 0.562, respectively (**Table 1**). Results of both Hancheng and Zhongqi showed that M medium could achieve the highest diversity of isolates, whereas it might activate more bacteria living from a certain environment than other media. Strains belonging to *Rhizobium, Staphylococcus, Bacillus, Methylobacterium*, and *Thauera* genera were isolated in all three media from Hancheng, whereas *Arthrobacter* and *Rhizobium* genera were isolated from Zhongqi using all three media (**Figure 4C**).

### Dominant Bacteria in Hancheng and Zhongqi

Among all isolates from the two coal samples, isolates of *Thauera*, *Arthrobacter*, and *Rhizobium* were the most prevalent. There were 224 *Thauera* isolates obtained from Hancheng coal with 32, 20, and 172 isolates obtained from M, W, and MW media, respectively. (**Figure 3A**; **Supplementary Table S4**). No *Thauera* isolates were identified from Zhongqi. These *Thauera* isolates were closely related to the species *Thauera phenylacetica* B4P (T), based on 16S rRNA gene sequence, with the highest similarities being 99.40%. Further BOX-PCR and REP-PCR analyses showed that there were 27 different patterns for *Thauera* (**Supplementary Table S5**). There were 309 *Arthrobacter* strains isolated from Zhongqi coal, including 7, 121, and 181 isolates from M, W, and MW media, respectively (**Figure 3A**). Among these, 117 and 179 isolates were closely related to *Arthrobacter defluvii* 4C1-a(T) and *A. oryzae* KV-651(T), with 16S rRNA gene sequence similarities being 99.50-99.57 and 98.50-100.00%, respectively (**Supplementary Table S2**). BOX-PCR and REP-PCR analyses showed 10 patterns of *Arthrobacter* (**Supplementary Table S6**). *Rhizobium* was the only genus that could be found in both coals and could be isolated using all media. A total of 246 *Rhizobium* isolates were isolated from Hancheng and Zhongqi, among which, 145 isolates from ZQM were closely related to *Rhizobium pusense* NRCPB10(T), with 16S rRNA gene sequence similarities being 99.80%; 76 isolates (70 isolates from HM, 1 isolate from HW, and 5 isolates from HMW) were closely related to *R. selenitireducens* B1(T) with 16S rRNA gene sequence similarities being 98.80-98.90%; 16 isolates (1 isolate from HM, 11 isolates from HW, 1 isolate from ZQM, 2 isolates from ZQW, and 1 isolate from ZQMW) were closely related to *R. massiliae* 90A, with 16S rRNA sequence similarities being 99.70-100.00%, and one isolate, only from ZQM, was closely related to *R. cellulosilyticum* ALA10B2(T), with 16 rRNA gene sequence similarity being 99.20%; two isolates from ZQM were closely related to *R. alkalisoli* CCBAU 01393(T), with 16S rRNA gene sequence similarities being 98.29%, and six isolates were closely related to *R. borbori* DN316(T) isolated from HW, with 16S rRNA gene sequence similarities being 99.69%. All *Rhizobium* isolates could be assigned to 12 patterns based on BOX-PCR and REP-PCR analyses (**Supplementary Table S7**).

### Lignin-Degrading Abilities

**FIGURE 5:**
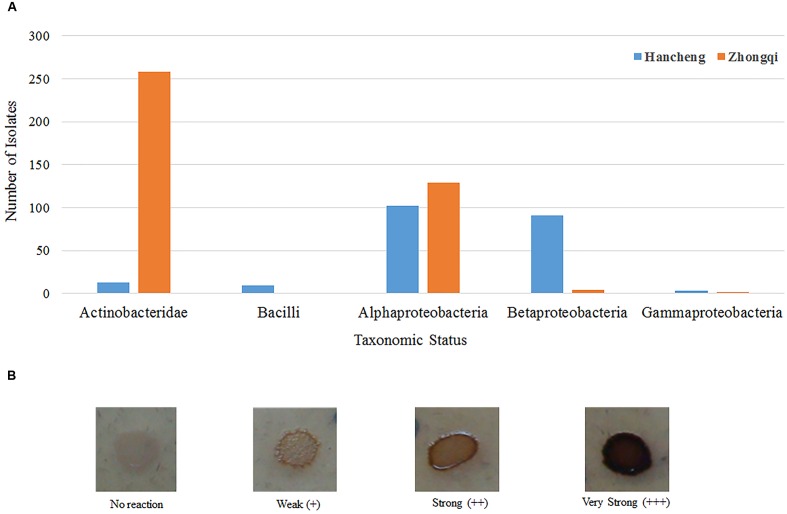
**Taxonomic distribution of different bacterial strains with lignin degradation abilities.** **(A)** Taxonomic distribution of different bacterial strains from Hancheng and Zhongqi. **(B)** Strains with the color reaction results indicating different degrees of lignin degradation ability.

Of all 876 isolates, 218 isolates (comprising 35 species) from Hancheng and 394 isolates (comprising 19 species) from Zhongqi showed positive results for lignin degradation, with varying abilities, on GU-WA agar plates (**Figure 5A**). Of these, 27, 335, and 250 isolates had very strong, strong, and weak lignin degradation ability, respectively (the color reaction results indicating different degrees of lignin degradation ability are shown in **Figure 5B**). The strains with very strong lignin degradation belonged to *Arthrobacter* (10/310 isolates), *Brachybacterium* (2/6 isolates), *Methylobacterium* (10/19 isolates), *Rhizobium* (1/246 isolate), and *Massilia* (4/5 isolates) (**Supplementary Table S4**). Genera having the highest number of lignin-degrading isolates were *Arthrobacter* (80.65% of *Arthrobacter* isolates) and *Thauera* (40.18% of *Thauera* isolates) in Hancheng and Zhongqi, respectively. Of the *Rhizobium* genus, 78.86% of isolates from both Hancheng and Zhongqi had lignin-degrading abilities (**Supplementary Table S4**).

From Hancheng, 90 of 224 isolates of *Thauera*, and 85 of 94 isolates of *Rhizobium* showed lignin-degrading abilities. From the remaining genera, 12 of 16 isolates of *Methylobacterium*, five of nine isolates of *Tessaracoccus*, four of five isolates of *Brachybacterium*, and four of eight isolates of *Bacillus* were positive for lignin degradation. In contrast, 255 of 309 isolates of *Arthrobacter* and 129 of 152 isolates of *Rhizobium*, from Zhongqi, had lignin-degrading abilities. From the remaining genera, four of five isolates of *Massilia* and two of two isolates of *Pseudomonas* had lignin degrading ability (**Supplementary Table S4**). Among all lignin degrading isolates, 233, 151, and 228 isolates were detected from M, W, and MW media, respectively. In contrast, lignin degradation abilities were not detected with isolates from the genera *Curtobacterium*, *Brevibacterium, Devosia, Brevundimonas*, and *Proteus*.

For *Arthrobacter*, 82.26% strains had lignin degradation ability. Among these functional strains, nine isolates had very strong lignin degradation abilities. These isolates were related to *A. chlorophenolicus* A6(T) (one isolate) and *A. oryzae* KV-651(T) (eight isolates), based on 16S rRNA gene similarities of 99.48% and 98.50–100.00%, respectively. For *Rhizobium*, 87.0% of isolates had lignin degradation ability. Among all 214 functional strains, one isolate was very strong for this function and was related to *R. massiliae* 90A, based on a 16S rRNA gene similarity of 100.00% (**Supplementary Table S2**). For *Thauera*, 40.17% strains had lignin degradation ability. However, none showed very strong lignin degradation ability.

### Detection of LMCO Gene

Along with the colony morphological characteristics, BOX-PCR, and REP-PCR analyses, 27, 12, and 10 *Thauera*, *Rhizobium*, and *Arthrobacter* isolates, covering all patterns, as well as 53 other strains representing separate clusters, were selected for detection the of LMCO genes.

**FIGURE 6:**
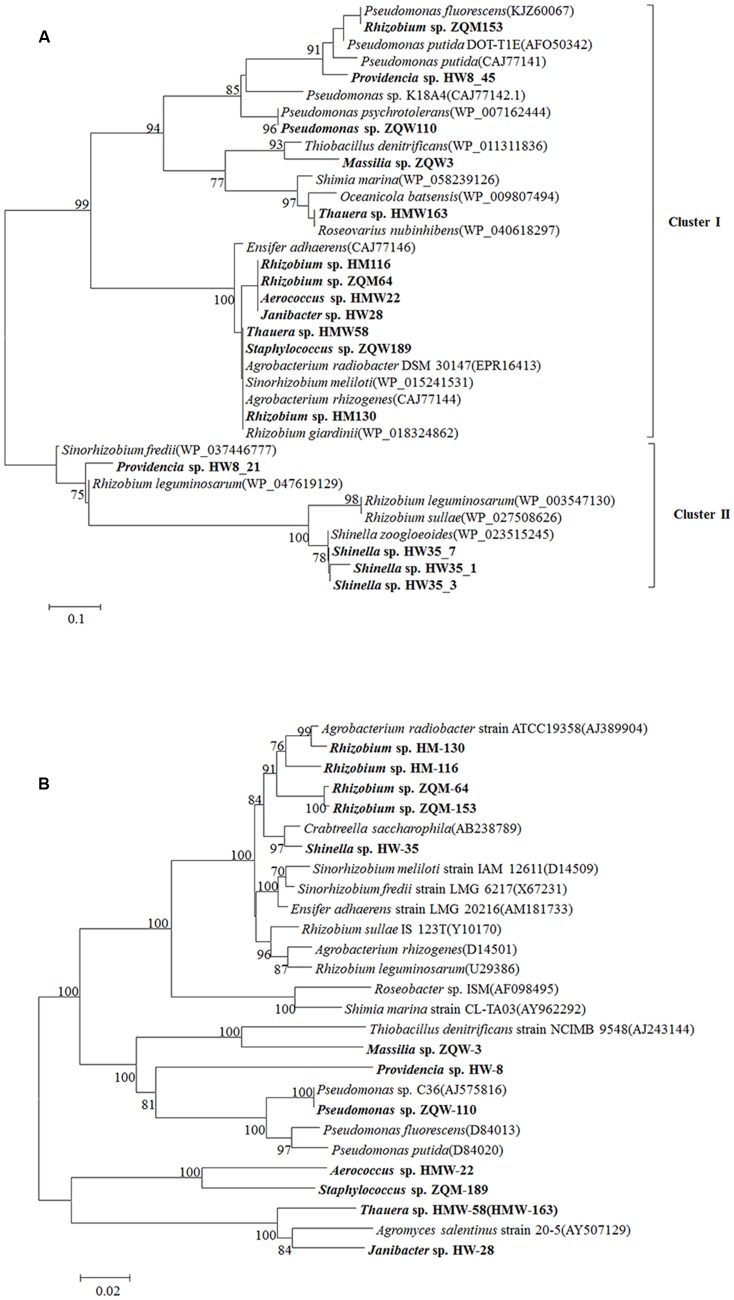
**Phylogenetic tree of LMCO genes detected from Hancheng and Zhongqi samples.** **(A)** LMCO genes based on the amino acid sequences. **(B)** The phylogenetic relationship based on the 16S rRNA gene in accordance to strains encoding LMCO gene from our study and their reference strains (using type strains for each species). Distance-based evolutionary trees were constructed by the neighbor-joining algorithm and 1,000 bootstrap replication using the MEGA 5.0 software. Bootstrap probabilities >70% are indicated at the branch nodes.

From 13 isolates, belonging to 10 genera of 102 original isolates, 16 LMCO genes with different amino acid sequences were detected (**Figure 6A**). This is the first report of LMCO genes discovered in the genera *Aerococcus, Shinella*, and *Massilia*. Phylogenetic analysis showed that these laccase genes were separated into two major clusters. Cluster I contained a mix LMCO genes from different taxa, such as *Rhizobium, Providencia, Pseudomonas, Massilia*, and *Thauera* from Proteobacteria, as well as *Aerococcus, Janibacter*, and *Staphylococcus*, from Actinobacteria and Firmicutes. Cluster II contained LMCO genes from *Providencia*, *Shinella*, *Sinorhizobium*, and *Rhizobium*. The topology of the LMCO gene based phylogenetic tree was much different from that of 16S rRNA gene-based phylogenetic tree (**Figures 6A,B**). For example, LMCO genes from *Rhizobium* were separated into different groups, whereas the 16S rRNA gene analysis resulted in these isolates being clustered together. The LMCO gene from *Rhizobium* sp. ZQM163 was closely related to that of *Pseudomonas* rather than *Rhizobium*. LMCO sequences from *Thauera* sp. HMW163 and *Thauera* sp. HMW58 were closely related to *Roseovarius* and *Agrobacterium*, respectively. However, the 16S rRNA gene sequences from these isolates shared 100.00% similarity and were closely related to *Agromyces*. Two LMCO genes were found in *Providencia* sp. HW8, one of which (HW8_45) was closely related to that from *Pseudomonas*, whereas the other (HW8_21) was closely related to that of *Rhizobium*. Moreover, genes in Cluster II, from both Proteobacteria and Actinobacteria, showed higher similarities (85.00–100.00%), which suggested that these genes might have been generated from the same ancestor. The results indicated that horizontal gene transfer of LMCO might happen frequently during these strains.

### Detection of LMCO Gene Expression

**FIGURE 7:**
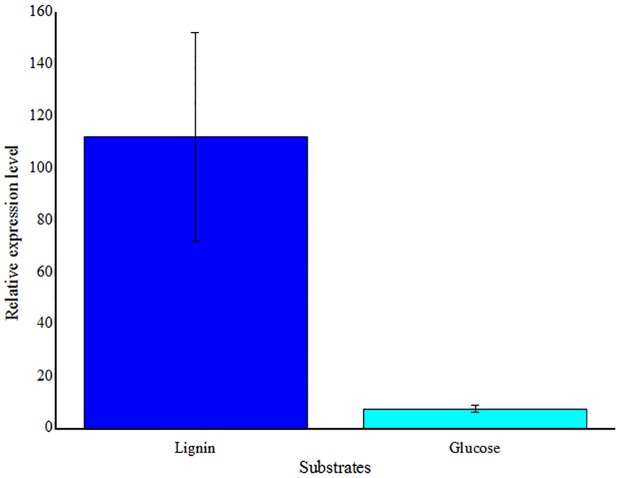
**Real-time RT-PCR analysis of LMCO gene transcription levels in *Massilia* sp. ZQW3 cells grown on lignin, compared to glucose control (*n* = 3/group).** All the data represent mean ± SD.

To detect the gene expression, one of stains that the LMCO were first detected was selected. *Massilia* sp. ZQW3 was chosed to detect LMCO gene expression. Expression levels were significantly upregulated with the stimulation of lignin, but in relative low levels when induced by glucose (**Figure 7**).

In summary, 876 strains were isolated from low and high rank coal of the Ordos Basin, using three kinds of media. Most isolates could degrade lignin, suggesting the potential for the application of bioconversion of coal. From 13 isolates, 16 LMCO genes with different amino acid sequences were detected. This is the first report of LMCO genes discovered in the genera *Aerococcus, Shinella*, and *Massilia*. High LMCO gene expression level was also detected in the genera *Massilia.* Moreover, LMCO genes were absent in most lignin degrading bacteria, suggesting the presence of other enzymes mediating lignin degradation. However, the indigenous microbial community of coal environments, especially the culturable strains, is still far from clear. Considering the poor culturability of oligotrophic bacteria, and the limitations of degenerate PCR approaches, more bacteria with novel genes and functions might be found in the future.

## Discussion

Although molecular methods allow for study of microbial ecosystems (Barns et al., 1994), some species of bacteria in the microbial community remain elusive, as they are unculturable. However, given the appropriate conditions they are able to grow. For biotechnical applications or understanding growth and life cycles, it is important to isolate pure cultures. Recently, microbial bioconversion of coal into soluble substances for the production of chemicals has attracted much attention. However, little has been known about the compositions and functions of endogenous microorganisms in coal. Tang et al. (2012) studied the diverse microbial community from the two coalbeds of the Ordos Basin in Hancheng and Bayannaoer. Herein, some strains isolated by cultivation were not detected by clone library analyses. For example, many isolates (such as strains that were closely related to *Shinella, Ancylobacter, Providencia* species and so on) belonging to Firmicutes, Alphaproteobacteria, and Actinobacteria from Hancheng coal sample, and isolates (such as strains that were closely related to *Micrococcus, Janibacter, Brevibacterium, Curtobacterium* species and so on) belonging to Firmicutes, Betaproteobacteria, and Alphaproteobacteria from Zhongqi coal sample were not detected by the clone library analyses (Tang et al., 2012; **Supplementary Figure S1**). Interestingly, the predominant bacteria of *Thauera* were detected only by a culture-independent method. It was suggested that the strains, which were only detected by a culture-dependent method, were so rare in the coal environment that their 16S rRNA genes could not be detected in clone library analyses. In the given medium/habitat, with limited matter and energy supplies, if some strains could grow well, others would be restricted. Therefore, the strains that were only detected by a culture-dependent method were able to survive and were easily cultured, indicating that special media could be used to screen these strains directly. In contrast, the common predominant bacteria detected with both culture-independent and -dependent methods was *Arthrobacter*, which was detected by clone library analyses in the proportion of 11.0% (Tang et al., 2012), and by the culture-dependent analyses in the proportion of 35.4%.

From other worldwide coal basins, culture-independent analysis (**Supplementary Figure S1**) based on 16S rRNA gene sequencing has readily identified the presence of Proteobacteria (Beckmann et al., 2011; Stepniewska et al., 2014), wherein our study, using special culture conditions, identified isolates belonging to the phyla Actinobacteria (such as strains that were closely related to *Tessaracoccus, Brevibacterium, Brachybacterium, Terrabacter, Micrococcus, Arthrobacter, Janibacter* species and so on) and Firmicutes (such as strains that were closely related to *Aerococcus, Bacillus, Staphylococcus, Lysinibacillus* species and so on).

Similar conditions enrich similar microorganisms, while different environments diversify the microbial community. In general, M medium contained soluble chemicals from coal, including organic substances and inorganic substances. W medium mainly contained inorganic substances, and MW medium contained both abundant organic and inorganic substances. For different media, the Shannon-Weiner index showed that the diversity of M was higher than that of the other two media for regions tested (**Table 1**). Thus, M media, being more similar to coal reservoir conditions than W and MW, can reveal more information about culturable bacteria from coal. In contrast, the diversity of MW medium was less than that of M and W media; however, we were able to obtain more isolates from MW medium, compared to M and W media, for both regions (**Table 1**). *Thauera* and *Arthrobacter* were the dominant strains identified from Hancheng and Zhongqi, respectively. Isolates of *Thauera* were discovered in the proportion of 23.7, 34.9, and 89.6% from HM, HW, and HMW, respectively. Similarly, isolates of *Arthrobacter* were identified in the proportion of 4.1, 91.7, and 99.5% from ZQM, ZQW, and ZQMW, respectively. The higher nutritional levels might have caused extensive growth of several dominant strains, and thus increased the number of isolates, while decreasing the diversity of isolates.

Although both coal samples were from Ordos Basin, only six bacterial genera were common to both (**Figure 4B**). In contrast, five and 16 genera were unique in Hancheng and Zhongqi coal, respectively. The discrepancy of bacterial isolates between the two coals (**Figures 2A,B**) indicated that the environment could select for different microorganisms or prompt evolution to adapt to the specific conditions. Actually, there were significant differences between the two coal samples. Hancheng coal belonged to meager lean coal, formed from marine-continental interactive sedimentation, during the Carboniferous-Permian period. It offered good preservation conditions for high rank coal-bed methane (CBM). In contrast, Zhongqi coal was brown coal from continental sedimentation formed in the middle and lower Jurassic period, and thus was younger than that of Hancheng by almost a 100 million years. Hancheng and Zhongqi coals had 9.6-11.0 and 35.5-49.0% volatile matter contents, respectively (Tang et al., 2012).

The influence of coal properties on microbial composition is not entirely clear and needs to be further researched; however, the trends of dominant isolates might give some clues. *Thauera* has been widely detected in marine sediments and soil (Etchebehere and Tiedje, 2005), and is known to have the ability to degrade a wide variety of aromatic compounds, with oxygen or nitrate as a terminal electron acceptor (Mechichi et al., 2002). In addition, it can also facilitate denitrification (Etchebehere and Tiedje, 2005). In fact, *Thauera* was detected in the deep coal seam groundwater of northern Japan (Shimizu et al., 2007), as well as from water samples from Australian coal seams (Li et al., 2008). In our study, *Thauera* was only isolated from Hancheng coal, which was sampled from a deeper well with an older sedimentation history, with a higher grade of maturity than Zhongqi coal. It had less alkane hydrocarbons and more aromatic hydrocarbons, which might have enriched for bacteria with aromatic hydrocarbon degradation abilities, such as *Thauera.*

*Arthrobacter* was isolated and detected from various coal related sites. For example, this genus was isolated from the spoil of brown coal colliery substrate, and comprised approximately 10% of the total strains isolated (Krištůfek et al., 2005). They were also detected from Canadian subsurface coal beds by a culture-independent method (Penner et al., 2010), and from the maturation of overburden, which has been disturbed by surface mining for 37 years, in southeastern Ohio, USA, but was not detected from undisturbed soil in the same region (Poncelet et al., 2014). Interestingly, *Arthrobacter* was frequently found to be present on plant foliage (Scheublin and Leveau, 2013). In our study, *Arthrobacter* was isolated from both Hancheng and Zhongqi, indicating it could survive in different habitats.

*Rhizobium* species were isolated from the coal bed environment from both Hancheng (94 isolates) and Zhongqi (152 isolates). Actually, *Rhizobium* species are widely distributed in nature and are usually isolated from the soil or aquatic environments, especially in the plant rhizosphere (Yoon et al., 2010; Sun et al., 2013). Recently, some strains were reported to be capable of utilizing aromatic compounds (Zhang et al., 2012). In 2010, a pot experiment was conducted to investigate the effects of *Glomus mosseae* and *Rhizobium* on *Medicago sativa* grown on three types of coal mine substrates (Wu et al., 2009). In 2011, the first occurrence of *Rhizobium* and *Chelatococcus* strains in a low rank Indian coal bed was shown (Singh and Tripathi, 2011). In our study, we also isolated *Rhizobium* from both from a high (Hancheng) and low (Zhongqi) rank coal, in China, suggesting the universal distribution of *Rhizobium* in coal.

Lignin and its derivatives are a major component of coal. Coal lignin is an important organic substance. In our study, 55.47 and 81.27% strains isolated from Hancheng and Zhongqi, respectively, have lignin degradation ability. Sixteen sequences of laccase genes from 13 isolates were detected, among which LMCO genes in the genera *Aerococcus, Shinella*, and *Massilia* were detected for the first time. High LMCO gene expression level was detected with lignin as sole carbon source, indicating its metabolic activity related to lignin degradation. All these findings have expanded the knowledge about the diversity of these LMCO genes in bacteria. It is interesting that many more isolates had lignin-degrading activity, based on the assay assessing color changes on GU-WA medium, compared with only 16 LMCO genes were identified in 13 isolates (**Figure 6A**). For example, many strains with lignin degradation functions from the genera of *Arthrobacter, Brachybacterium, Leucobacter, Terrabacter, Tessaracoccus, Lysinibacillus, Bacillus, Bosea, Methylobacterium, Ancylobacter, Diaphorobacter*, and *Acinetobacter* had no detectable LMCO gene. The results suggested the presence of unknown laccase enzymes or other enzymes that mediate lignin degradation.

Furthermore, when the LMCO gene phylogenetic tree (**Figure 6A**) was compared with the 16S rRNA gene phylogenetic tree (**Figure 6B**), it was observed that stains having similar LMCO genes could be divergent based on 16S rRNA gene sequences. It was suggested that a high rate of horizontal gene transfer of LMCO genes might have occurred in these strains isolated from coal.

## Author Contributions

X-LW is the main instructor. LW did the main experiments and wrote this paper. YN, Y-QT, and X-MS also helped to instruct the experiment. KC, L-ZS, and Z-JW helped to exploit samples and do some experiments.

## Conflict of Interest Statement

The authors declare that the research was conducted in the absence of any commercial or financial relationships that could be construed as a potential conflict of interest.

## Footnotes

1 http://blast.ncbi.nlm.nih.gov/Blast.cgi
