# Supplementary material for: Diverse Bacteria with Lignin Degrading Potentials Isolated from Two Ranks of Coal
Source: Front Microbiol. 2016 Sep 9;7:1428. doi: 10.3389/fmicb.2016.01428 (PMC5016517; doi:10.3389/fmicb.2016.01428)
Supplement: Supplementary file 1 [file Data_Sheet_1.DOCX]

**Diverse bacteria with lignin degrading potentials isolated from two ranks of coal**

***Lu Wang****^1^****, Yong Nie****^2^****, Yue-Qin Tang****^3^****, Xin-Min Song****^4^****, Kun Cao****^5^****, Li-zhu Sun****^5^****, Zhi-jian Wang****^5^****, and Xiao-Lei Wu****^2^********

**Supplementary Table 1.** Primer sequences used in this study.

**Supplementary Table 2.** The 16S rRNA gene similarities of the isolates to known type strains.

**Supplementary Table 3.** The GenBank accession numbers for the LMCO gene sequences of the strains isolated in this study.

**Supplementary Table 4.** Classification of bacteria isolated from coals with their LMCO genes and their lignin degradation ability.

**Supplementary Table 5.** Classification of 145 *Thauera* isolates based on BOX-PCR and REP-PCR genotyping groups and distribution of LMCO genes.

**Supplementary Table 6.** Classification of 309 *Arthrobacter* isolates based on BOX-PCR and REP-PCR genotyping groups and distribution of LMCO genes.

**Supplementary Table 7.** Classification of 246 *Rhizobium* isolates based on BOX-PCR and REP-PCR genotyping groups and distribution of LMCO genes.

**Supplementary Figure 1.** Phylogenetic tree showing the genetic relationships. Comparison PCR products of 16S rRNA genes of the representative isolates from Hancheng and Zhongqi in the Ordos Basin, with 16S rRNA genes from coals that reported in the world including Ordos Basin, which were determined by denaturing gradient gel electrophoresis (DGGE) (Nicomrat et al., 2006; Beckmann et al., 2011), clone library (Shimizu et al., 2007; Penner et al., 2010; Singh et al., 2011; Ohtomo et al., 2013) and PCR products (Stepniewska et al., 2014) analysis. All the sequences were aligned and analyzed by the neighbor-joining method using MEGA 5.0. The trees were bootstrapped with 1000 replicates.

**Supplementary Table 1.**

| **Genes** | **Primers** | **Sequences 5’ to 3’** | **Legth (bp)** | **Annealing temp (℃)** |
| --- | --- | --- | --- | --- |
| 16S rRNA gene (Wang et al. 2007) | 8F | AGAGTTTGATCCTGGCTCAG | 1500 | 55 |
|  | 1492R | GGTTACCTTGTTACGACTT |  |  |
| LMCO gene (Kellner H. 2008) | Cu1AF | ACMWCBGTYCAYTGGCAYGG | 100 | 50 |
|  | Cu2R | GRCTGTGGTACCAGAANGTNCC |  |  |
| BOX-PCR (Versalovic et al., 1994) | BOX A1R | CTACGGCAAGGCGACGCTGACG | 57 | 53 |
| REP-PCR (Doll et al., 1993) | GTG_5_ | GTGGTGGTGGTGGTG | 35-40 | 45 |

**Supplementary Table 2.**

| **Isolate**  **(No. of isolates)** | **16S rDNA accession No.** | **Closest type strain** | **16S rDNA similarity (%)** | |
| --- | --- | --- | --- | --- |
|  |  |  |  |  |
| HM116 (70) | KP152611 | *Rhizobium selenitireducens* B1(T) (EF440185) | 98.80 % | |
| HM39 (1) | KP152610 | *Rhizobium massiliae* 90A (AF531767) | 99.90 % | |
| HM198 (1) | KP152608 | *Devosia insulae* DS-56(T) (EF012357) | 100.00 % | |
| HM48 (1) | KP152607 | *Bosea vestrisii* 34635(T) (AF288306) | 99.00 % | |
| HM1 (1) | KP152609 | *Methylobacterium aminovorans* JCM 8240(T) (AB175629) | 99.00 % | |
| HM88 (1) | KP152606 | *Bacillus halodurans* ATCC 27557(T) (AB021187) | 99.90 % | |
| HM50 (1) | KP152599 | *Brevibacterium halotolerans* DSM 8802(T) (AM747812) | 100.00 % | |
| HM66 (2) | KP152601 | *Staphylococcus haemolyticus* ATCC 29970(T) (L37600) | 99.78 % | |
| HM90 (1) | KP152600 | *Lysinibacillus macroides* LMG 18474(T) (AJ628749) | 99.70 % | |
| HM33 (1) | KP152602 | *Bacillus cereus* ATCC 14579(T) (AE016877) | 99.90 % | |
| HM83 (1) | KP152605 | *Bacillus aquimaris* TF-12(T) (AF483625) | 99.00 % | |
| HM74 (1) | KP152604 | *Bacillus halosaccharovorans* E33(T) (NR_109116) | 98.97 % | |
| HM46 (1) | KP152603 | *Bacillus niacini* IFO 15566(T) (AB021194) | 99.70 % | |
| HM4 (1) | KP152613 | *Acinetobacter lwoffii* DSM 2403(T) (X81665) | 99.00 % | |
| HM16 (32) | KP152612 | *Thauera phenylacetica* B4P(T) (AJ315678) | 99.00 % | |
| HM19 (4) | KP152594 | *Brachybacterium paraconglomeratum* LMG 19861(T) (AJ415377) | 100.00 % | |
| HM35 (1) | KP152598 | *Brevibacterium sanguinis* CF63(T) (AJ564859) | 100.00 % | |
| HM37 (1) | KP152593 | *Arthrobacter nitroguajacolicus* G2-1(T) (AJ512504) | 100.00 % | |
| HM164 (3) | KP152595 | *Janibacter terrae* CS12(T) (AF176948) | 99.90 % | |
| HM5 (1) | KP152596 | *Micrococcus endophyticus* YIM 56238(T) (EU005372) | 99.00 % | |
| HM8 (9) | KP152597 | *Tessaracoccus lapidicaptus* IPBSL-7(T) (NR_134214) | 100.00 % | |
| HW1 (11) | KP152649 | *Rhizobium massiliae* 90A (AF531767) | 99.92 % | |
| HW30 (1) | KP152650 | *Rhizobium selenitireducens* B1(T) (EF440185) | 98.80 % | |
| HW2 (6) | KP152651 | *Rhizobium borbori* DN316(T) (EF125187) | 99.69 % | |
| HW35 (3) | KP152652 | *Shinella granuli* Ch06(T) (AY995149) | 99.10 % | |
| HW7 (2) | KP152653 | *Ancylobacter rudongensis* AS1.1761(T) (AY056830) | 99.71 % | |
| HW9 (13) | KP152648 | *Methylobacterium aminovorans* JCM 8240(T) (AB175629) | 100.00 % | |
| HW8 (2) | KP152657 | *Providencia vermicola* OP1(T) (AM040495) | 100.00 % | |
| HW16 (1) | KP152656 | *Proteus mirabilis* ATCC 29906(T) (ACLE01000013) | 99.80 % | |
| HW37 (20) | KP152655 | *Thauera phenylacetica* B4P(T) (AJ315678) | 99.00 % | |
| HW12 (3) | KP152654 | *Diaphorobacter nitroreducens* NA10B(T) (AB064317) | 99.90 % | |
| HW28 (2) | KP152645 | *Janibacter terrae* CS12(T) (AF176948) | 99.80 % | |
| HW4 (1) | KP152647 | *Bacillus barbaricus* V2-BIII-A2(T) (AJ422145) | 99.30 % | |
| HW5 (1) | KP152646 | *Staphylococcus epidermidis* ATCC 14990(T) (L37605) | 99.90 % | |
| HMW3 (172) | KP152590 | *Thauera phenylacetica* B4P(T) (AJ315678) | 99.40 % | |
| HMW59 (1) | KP152591 | *Acinetobacter radioresistens* DSM 6976(T) (X81666) | 99.90 % | |
| HMW30 (1) | KP152592 | *Acinetobacter lwoffii* DSM 2403(T) (X81665) | 99.80 % | |
| HMW78 (2) | KP152587 | *Methylobacterium aminovorans* JCM 8240(T) (AB175629) | 100.00 % | |
| HMW180 (1) | KP152589 | *Ancylobacter rudongensis* AS1.1761(T) (AY056830) | 99.70 % | |
| HMW60 (5) | KP152588 | *Rhizobium selenitireducens* B1(T) (EF440185) | 98.90 % | |
| HMW23 (1) | KP152581 | *Brachybacterium paraconglomeratum* LMG 19861(T) (AJ415377) | 99.70 % | |
| HMW58 (1) | KP152583 | *Micrococcus yunnanensis* YIM 65004(T) (FJ214355) | 99.70 % | |
| HMW6 (1) | KP152582 | *Leucobacter aridicollis* CIP 108388(T) (AJ781047) | 100.00 % | |
| HMW22 (1) | KP152586 | *Aerococcus urinaeequi* IFO 12173 (D87677) | 99.90 % | |
| HMW126 (5) | KP152584 | *Staphylococcus hominis subsp. novobiosepticus* GTC 1228(T) (AB233326) | 99.80 % | |
| HMW151 (1) | KP152585 | *Bacillus thuringiensis* ATCC 10792(T) (ACNF01000156) | 99.90 % | |
| ZQM19 (1) | KP152640 | *Rhizobium pusense* NRCPB10(T) (FJ969841) | 99.80 % | |
| ZQM77 (145) | KP152641 | *Rhizobium massiliae* 90A (AF531767) | 99.70 % | |
| ZQM64 (1) | KP152642 | *Rhizobium cellulosilyticum* ALA10B2(T) (DQ855276) | 99.20 % | |
| ZQM153 (2) | KP152643 | *Rhizobium alkalisoli* CCBAU 01393(T) (EU074168) | 98.29 % | |
| ZQM218 (1) | KP152637 | *Brevundimonas diminuta* ATCC 11568(T) (GL883089) | 99.77 % | |
| ZQM201 (1) | KP152638 | *Methylobacterium tardum* RB677(T) (AB252208) | 99.30 % | |
| ZQM1 (2) | KP152639 | *Methylobacterium thiocyanatum* DSM 11490(T) (AB175646) | 99.60 % | |
| ZQM176 (1) | KP152644 | *Pseudomonas baetica* a390(T) (FM201274) | 99.60 % | |
| ZQM189 (1) | KP152635 | *Staphylococcus cohnii subsp. cohnii* ATCC 29974(T) (D83361) | 100.00 % | |
| ZQM167 (2) | KP152636 | *Staphylococcus cohnii subsp. urealyticus* ATCC 49330(T) (AB009936) | 100.00 % | |
| ZQM184 (1) | KP152633 | *Curtobacterium flaccumfaciens* LMG 3645(T) (AJ312209) | 100.00 % | |
| ZQM38 (3) | KP152634 | *Terrabacter lapilli* LR-26(T) (AM690744) | 99.77 % | |
| ZQM207 (1) | KP152632 | *Brachybacterium paraconglomeratum* LMG 19861(T) (AJ415377) | 99.70 % | |
| ZQM109 (2) | KP152630 | *Arthrobacter defluvii* 4C1-a(T) (AM409361) | 99.50 % | |
| ZQM172 (5) | KP152631 | *Arthrobacter oryzae* KV-651(T) (AB279889) | 98.50 % | |
| ZQW4 (1) | KP152614 | *Arthrobacter oryzae* KV-651(T) (AB279889) | 98.60 % | |
| ZQW135 (2) | KP152615 | *Arthrobacter oryzae* KV-651(T) (AB279889) | 100.00 % | |
| ZQW102 (117) | KP152616 | *Arthrobacter defluvii* 4C1-a(T) (AM409361) | 99.57 % | |
| ZQW136 (1) | KP152617 | *Arthrobacter pascens* DSM 20545(T) (X80740) | 98.29 % | |
| ZQW9 (1) | KP152618 | *Micoccus yunnanensis* YIM 65004(T) (FJ214355) | 99.60 % | |
| ZQW38 (1) | KP152619 | *Terrabacter lapilli* LR-26(T) (AM690744) | 99.70 % | |
| ZQW70 (1) | KP152620 | *Staphylococcus epidermidis* ATCC 14990(T) (L37605) | 99.90 % | |
| ZQW93 (2) | KP152621 | *Rhizobium massiliae* 90A (AF531767) | 100.00 % | |
| ZQW110 (1) | KP152625 | *Pseudomonas psychrotolerans* C36(T) (AJ575816) | 99.90 % | |
| ZQW60 (1) | KP152622 | *Massilia aerilata* 5516S-11(T) (EF688526) | 98.59 % | |
| ZQW1 (3) | KP152623 | *Massilia suwonensis* 5414S-25(T) (FJ969487) | 98.00% | |
| ZQW3 (1) | KP152624 | *Massilia kyonggiensis* TSA1 (T) (NR_126273) | 98.00 % | |
| ZQMW1 (179) | KP152626 | *Arthrobacter oryzae* KV-651(T) (AB279889) | 98.57 % | |
| ZQMW103 (1) | KP152627 | *Arthrobacter pascens* DSM 20545(T) (X80740) | 98.35 % | |
| ZQMW8 (1) | KP152628 | *Arthrobacter chlorophenolicus* A6(T) (CP001341) | | 99.48 % |
| ZQMW86 (1) | KP152629 | *Rhizobium massiliae* 90A (AF531767) | | 100.00 % |

**Supplementary Table 3.**

| **Isolates** | **LMCO accession No.** |
| --- | --- |
| HM116 | KX440098 |
| HM130 | KX440099 |
| HMW22 | KX440100 |
| HMW58 | KX440101 |
| HMW163 | KX440102 |
| HW8_21 | KX440103 |
| HW8_45 | KX440104 |
| HW28 | KX440105 |
| HW35_1 | KX440106 |
| HW35_3 | KX440107 |
| HW35_7 | KX440108 |
| ZQM64 | KX440109 |
| ZQM153 | KX440110 |
| ZQW3 | KX440111 |
| ZQW110 | KX440112 |
| ZQM189 | KX440113 |

**Supplementary Table 4.**

|  | **HM-16S rDNA** | | | **HM-Genes** | **HM-GU-WA oxidation^e^** | **HW-16S rDNA** | | | **HW-Genes** | **HW-GU-WA oxidation** | **HMW-16S rDNA** | | | **HMW-Genes** | **HMW-GU-WA oxidation** |
| --- | --- | --- | --- | --- | --- | --- | --- | --- | --- | --- | --- | --- | --- | --- | --- |
|  | **Num of S^a^** | **Num of I^b^** | **rate^c^ of HM(%)** | **LMCO^d^** |  | **Num of S** | **Num of I** | **rate of HW(%)** | **LMCO** |  | **Num of S** | **Num of I** | **rate of HMW(%)** | **LMCO** |  |
| *Arthrobacter* | 1 | 1 | 0.74 | 0 | 0 | 0 | 0 | 0 | 0 | 0 | 0 | 0 | 0 | 0 | 0 |
| *Brachybacterium* | 1 | 4 | 2.96 | 0 | 1(3) | 0 | 0 | 0 | 0 | 0 | 1 | 1 | 0.52 | 0 | 1(1+++) |
| *Curtobacterium* | 0 | 0 | 0 | 0 | 0 | 0 | 0 | 0 | 0 | 0 | 0 | 0 | 0 | 0 | 0 |
| *Janibacter* | 1 | 3 | 2.22 | 0 | 1(1) | 1 | 2 | 3.03 | 1(1-1) | 1(1++) | 0 | 0 | 0 | 0 | 0 |
| *Leucobacter* | 0 | 0 | 0 | 0 | 0 | 0 | 0 | 0 | 0 | 0 | 1 | 1 | 0.52 | 0 | 1(1+) |
| *Micrococcus* | 1 | 1 | 0.74 | 0 | 1(1) | 0 | 0 | 0 | 0 | 0 | 1 | 1 | 0.52 | 1(1-1) | 0 |
| *Terrabacter* | 0 | 0 | 0 | 0 | 0 | 0 | 0 | 0 | 0 | 0 | 0 | 0 | 0 | 0 | 0 |
| *Tessaracoccus* | 1 | 9 | 6.67 | 0 | 1(5,+) | 0 | 0 | 0 | 0 | 0 | 0 | 0 | 0 | 0 | 0 |
| *Brevibacterium* | 1 | 1 | 0.74 | 0 | 0 | 0 | 0 | 0 | 0 | 0 | 0 | 0 | 0 | 0 | 0 |
| **Actinobacteridae** | **6** | **19** | **14.07** | **0** | **4** | **1** | **2** | **3.03** | **0** | **1** | **3** | **3** | **1.56** | **1(1-1)** | **2** |
| *Lysinibacillus* | 1 | 1 | 0.74 | 0 | 1(1) | 0 | 0 | 0 | 0 | 0 | 0 | 0 | 0 | 0 | 0 |
| *Staphylococcus* | 1 | 2 | 1.48 | 0 | 0 | 1 | 1 | 1.52 | 0 | 1(1++) | 1 | 5 | 2.6 | 0 | 1(2++) |
| *Bacillus* | 6 | 6 | 4.44 | 0 | 2(2 +) | 1 | 1 | 1.52 | 0 | 1(1++) | 1 | 1 | 0.52 | 0 | 1(1+) |
| *Aerococcus* | 0 | 0 | 0 | 0 | 0 | 0 | 0 | 0 | 0 | 0 | 1 | 1 | 0.52 | 1(1-1) | 1(1++) |
| **Firmicutes** | **8** | **9** | **6.67** | **0** | **3** | **2** | **2** | **3.03** | **0** | **2** | **3** | **7** | **3.65** | **1(1-1)** | **3** |
| *Bosea* | 1 | 1 | 0.74 | 0 | 1(1+) | 0 | 0 | 0 | 0 | 0 | 0 | 0 | 0 | 0 | 0 |
| *Devosia* | 1 | 1 | 0.74 | 0 | 0 | 0 | 0 | 0 | 0 | 0 | 0 | 0 | 0 | 0 | 0 |
| *Brevundimonas* | 0 | 0 | 0 | 0 | 0 | 0 | 0 | 0 | 0 | 0 | 0 | 0 | 0 | 0 | 0 |
| *Methylobacterium* | 1 | 1 | 0.74 | 0 | 1(1+++) | 1 | 13 | 19.7 | 0 | 1(9+++) | 1 | 2 | 1.04 | 0 | 1(2++) |
| *Rhizobium* | 2 | 71 | 52.59 | 2(2-2) | 2(59++,8+) | 3 | 18 | 27.27 | 0 | 2(13++) | 1 | 5 | 2.6 | 0 | 1(5++) |
| *Shinella* | 0 | 0 | 0 | 0 | 0 | 1 | 3 | 4.55 | 1(1-3) | 1(1++) | 0 | 0 | 0 | 0 | 0 |
| *Ancylobacter* | 0 | 0 | 0 | 0 | 0 | 1 | 2 | 3.03 | 0 | 1(2++) | 1 | 1 | 0.52 | 0 | 1(1++) |
| **Alphaproteobacteria** | **5** | **74** | **54.81** | **2(2-2)** | **4** | **6** | **36** | **54.55** | **0** | **5** | **3** | **8** | **4.17** | **0** | **3** |
| *Massilia* | 0 | 0 | 0 | 0 | 0 | 0 | 0 | 0 | 0 | 0 | 0 | 0 | 0 | 0 | 0 |
| *Diaphorobacter* | 0 | 0 | 0 | 0 | 0 | 1 | 3 | 4.55 | 0 | 1(1++) | 0 | 0 | 0 | 0 | 0 |
| *Thauera* | 1 | 32 | 23.7 | 0 | 1(15+) | 1 | 20 | 30.3 | 0 | 2(18++) | 1 | 172 | 89.58 | 1(1-1) | 1(57++) |
| **Betaproteobacteria** | **1** | **32** | **23.7** | **0** | **1** | **2** | **23** | **34.85** | **0** | **3** | **1** | **172** | **89.58** | **1(1-1)** | **1** |
| *Acinetobacter* | 1 | 1 | 0.74 | 0 | 0 | 0 | 0 | 0 | 0 | 0 | 2 | 2 | 1.04 | 0 | 2(2++) |
| *Pseudomonas* | 0 | 0 | 0 | 0 | 0 | 0 | 0 | 0 | 0 | 0 | 0 | 0 | 0 | 0 | 0 |
| *Proteus* | 0 | 0 | 0 | 0 | 0 | 1 | 1 | 1.52 | 0 | 0 | 0 | 0 | 0 | 0 | 0 |
| *Providencia* | 0 | 0 | 0 | 0 | 0 | 1 | 2 | 3.03 | 1(1-2) | 1(1++) | 0 | 0 | 0 | 0 | 0 |
| **Gammaproteobacteria** | **1** | **1** | **0.74** | **0** | **0** | **2** | **3** | **4.55** | **1(1-2)** | **1** | **2** | **2** | **1.04** | **0** | **2** |
| **Proteobacteria** | **7** | **107** | **79.25** | **2(2-2)** | **5** | **10** | **62** | **93.95** | **1(1-2)** | **9** | **6** | **182** | **94.79** | **1(1-1)** | **6** |
| **Total number** | **21** | **135** | **1** | **2(2-2)** | **12** | **13** | **66** | **1** | **3(3-6)** | **12** | **12** | **192** | **1** | **3(3-3)** | **11** |

|  | **ZQM-16S rDNA** | | | **ZQM-Genes** | **ZQM-GU-WA oxidation** | **ZQW-16S rDNA** | | | **ZQW-Genes** | **ZQW-GU-WA oxidation** | **ZQMW-16S rDNA** | | | **ZQMW-Genes** | **ZQMW-GU-WA oxidation** |
| --- | --- | --- | --- | --- | --- | --- | --- | --- | --- | --- | --- | --- | --- | --- | --- |
|  | **Num of S** | **Num of I** | **rate of ZQM(%)** | **LMCO** |  | **Num of S** | **Num of I** | **rate of ZQW(%)** | **LMCO** |  | **Num of S** | **Num of I** | **rate of ZQMW(%)** | **LMCO** |  |
| *Arthrobacter* | 2 | 7 | 4.14 | 0 | 2(5++) | 3 | 121 | 91.7 | 0 | 2(53++,43+) | 3 | 181 | 99.5 | 0 | 3(9+++,55++,90+) |
| *Brachybacterium* | 1 | 1 | 0.59 | 0 | 1(1+++) | 0 | 0 | 0 | 0 | 0 | 0 | 0 | 0 | 0 | 0 |
| *Curtobacterium* | 1 | 1 | 0.59 | 0 | 0 | 0 | 0 | 0 | 0 | 0 | 0 | 0 | 0 | 0 | 0 |
| *Janibacter* | 0 | 0 | 0 | 0 | 0 | 0 | 0 | 0 | 0 | 0 | 0 | 0 | 0 | 0 | 0 |
| *Leucobacter* | 0 | 0 | 0 | 0 | 0 | 0 | 0 | 0 | 0 | 0 | 0 | 0 | 0 | 0 | 0 |
| *Micrococcus* | 0 | 0 | 0 | 0 | 0 | 1 | 1 | 0.76 | 0 | 1(1++) | 0 | 0 | 0 | 0 | 0 |
| *Terrabacter* | 1 | 3 | 1.78 | 0 | 1(1++) | 1 | 1 | 0.76 | 0 | 0 | 0 | 0 | 0 | 0 | 0 |
| *Tessaracoccus* | 0 | 0 | 0 | 0 | 0 | 0 | 0 | 0 | 0 | 0 | 0 | 0 | 0 | 0 | 0 |
| *Brevibacterium* | 0 | 0 | 0 | 0 | 0 | 0 | 0 | 0 | 0 | 0 | 0 | 0 | 0 | 0 | 0 |
| **Actinobacteridae** | **5** | **12** | **7.1** | **0** | **4** | **5** | **123** | **93.2** | **0** | **3** | **3** | **181** | **99.5** | **0** | **3** |
| *Lysinibacillus* | 0 | 0 | 0 | 0 | 0 | 0 | 0 | 0 | 0 | 0 | 0 | 0 | 0 | 0 | 0 |
| *Staphylococcus* | 2 | 3 | 1.78 | 1(1-1) | 1(1++) | 1 | 1 | 0.76 | 0 | 0 | 0 | 0 | 0 | 0 | 0 |
| *Bacillus* | 0 | 0 | 0 | 0 | 0 | 0 | 0 | 0 | 0 | 0 | 0 | 0 | 0 | 0 | 0 |
| *Aerococcus* | 0 | 0 | 0 | 0 | 0 | 0 | 0 | 0 | 0 | 0 | 0 | 0 | 0 | 0 | 0 |
| **Firmicutes** | **2** | **3** | **1.78** | **1(1-1)** | **1** | **1** | **1** | **0.76** | **0** | **0** | **0** | **0** | **0** | **0** | **0** |
| *Bosea* | 0 | 0 | 0 | 0 | 0 | 0 | 0 | 0 | 0 | 0 | 0 | 0 | 0 | 0 | 0 |
| *Devosia* | 0 | 0 | 0 | 0 | 0 | 0 | 0 | 0 | 0 | 0 | 0 | 0 | 0 | 0 | 0 |
| *Brevundimonas* | 1 | 1 | 0.59 | 0 | 0 | 0 | 0 | 0 | 0 | 0 | 0 | 0 | 0 | 0 | 0 |
| *Methylobacterium* | 2 | 3 | 1.78 | 0 | 0 | 0 | 0 | 0 | 0 | 0 | 0 | 0 | 0 | 0 | 0 |
| *Rhizobium* | 4 | 149 | 88.2 | 2(2-2) | 2(37++,90+) | 1 | 2 | 1.52 | 0 | 1(1++) | 1 | 1 | 0.55 | 0 | 1(1+++) |
| *Shinella* | 0 | 0 | 0 | 0 | 0 | 0 | 0 | 0 | 0 | 0 | 0 | 0 | 0 | 0 | 0 |
| *Ancylobacter* | 0 | 0 | 0 | 0 | 0 | 0 | 0 | 0 | 0 | 0 | 0 | 0 | 0 | 0 | 0 |
| **Alphaproteobacteria** | **7** | **153** | **90.5** | **2(2-2)** | **2** | **1** | **2** | **1.52** | **0** | **1** | **1** | **1** | **0.55** | **0** | **1** |
| *Massilia* | 0 | 0 | 0 | 0 | 0 | 3 | 5 | 3.79 | 1(1-1) | 2(4+++) | 0 | 0 | 0 | 0 | 0 |
| *Diaphorobacter* | 0 | 0 | 0 | 0 | 0 | 0 | 0 | 0 | 0 | 0 | 0 | 0 | 0 | 0 | 0 |
| *Thauera* | 0 | 0 | 0 | 0 | 0 | 0 | 0 | 0 | 0 | 0 | 0 | 0 | 0 | 0 | 0 |
| **Betaproteobacteria** | **0** | **0** | **0** | **0** | **0** | **3** | **5** | **3.79** | **1(1-1)** | **2** | **0** | **0** | **0** | **0** | **0** |
| *Acinetobacter* | 0 | 0 | 0 | 0 | 0 | 0 | 0 | 0 | 0 | 0 | 0 | 0 | 0 | 0 | 0 |
| *Pseudomonas* | 1 | 1 | 0.59 | 0 | 1(1+) | 1 | 1 | 0.76 | 1(1-1) | 1(1+++) | 0 | 0 | 0 | 0 | 0 |
| *Proteus* | 0 | 0 | 0 | 0 | 0 | 0 | 0 | 0 | 0 | 0 | 0 | 0 | 0 | 0 | 0 |
| *Providencia* | 0 | 0 | 0 | 0 | 0 | 0 | 0 | 0 | 0 | 0 | 0 | 0 | 0 | 0 | 0 |
| **Gammaproteobacteria** | **1** | **1** | **0.59** | **0** | **1** | **1** | **1** | **0.76** | **1(1-1)** | **1** | **0** | **0** | **0** | **0** | **0** |
| **Proteobacteria** | **8** | **154** | **91.1** | **2(2-2)** | **3** | **5** | **8** | **6.07** | **2(2-2)** | **4** | **1** | **1** | **0.55** | **0** | **1** |
| **Total number** | **15** | **169** | **1** | **2(2-2)** | **8** | **11** | **132** | **1** | **3(3-3)** | **7** | **4** | **182** | **1** | **0** | **4** |

S^a^, the number of patterns;

I^b^, the number of isolates;

rate^c^, the isolates’ rate in corresponding sample;

LMCO^d^, for example, “X(Y-Z)”, X-the number of patterns, Y-the number of isolates, and Z-the number of gene sequences detected;

HM-GU-WA oxidation^e^, for example, “P(Q)”, P-the number of patterns, Q-the number of isolates; “++”, “+”, “(+)”, and “-” indicate the color circle changed degree from very strong to no reaction.

**Supplementary Table 5.**

| **No. of Pattern** | **Strains** | **LMCO gene** |
| --- | --- | --- |
| 1 | HMW150 | + |
| 2 | HMW106 | - |
| 3 | HM112 | + |
| 4 | HMW24 | + |
| 5 | HM108 | - |
| 6 | HMW57 | - |
| 7 | HMW17 | + |
| 8 | HMW86 | + |
| 9 | HMW90 | - |
| 10 | HMW168 | - |
| 11 | HMW118 | - |
| 12 | HMW14 | - |
| 13 | HMW71 | - |
| 14 | HMW94 | + |
| 15 | HMW163 | + |
| 16 | HMW110 | - |
| 17 | HMW193 | - |
| 18 | HMW58 | + |
| 19 | HM126 | + |
| 20 | HMW121 | - |
| 21 | HMW87 | + |
| 22 | HM194 | - |
| 23 | HMW169 | - |
| 24 | HMW55 | + |
| 25 | HM101 | + |
| 26 | HMW88 | - |
| 27 | HMW40 | - |

**Supplementary Table 6.**

| **No. of Pattern** | **Strains** | **LMCO gene** |
| --- | --- | --- |
| 1 | HM37 | - |
| 2 | ZQM109 | - |
| 3 | ZQM172 | - |
| 4 | ZQW4 | - |
| 5 | ZQW135 | - |
| 6 | ZQW102 | - |
| 7 | ZQW136 | - |
| 8 | ZQMW1 | - |
| 9 | ZQMW103 | - |
| 10 | ZQMW8 | - |

**Supplementary Table 7.**

| **No. of Pattern** | **Strains** | **LMCO gene** |
| --- | --- | --- |
| 1 | HM116 | + |
| 2 | HM39 | + |
| 3 | HW1 | - |
| 4 | HW30 | - |
| 5 | HW2 | - |
| 6 | HMW60 | - |
| 7 | ZQM19 | - |
| 8 | ZQM77 | - |
| 9 | ZQM64 | + |
| 10 | ZQM153 | + |
| 11 | ZQW93 | - |
| 12 | ZQMW86 | - |

**Supplementary Figure 1.
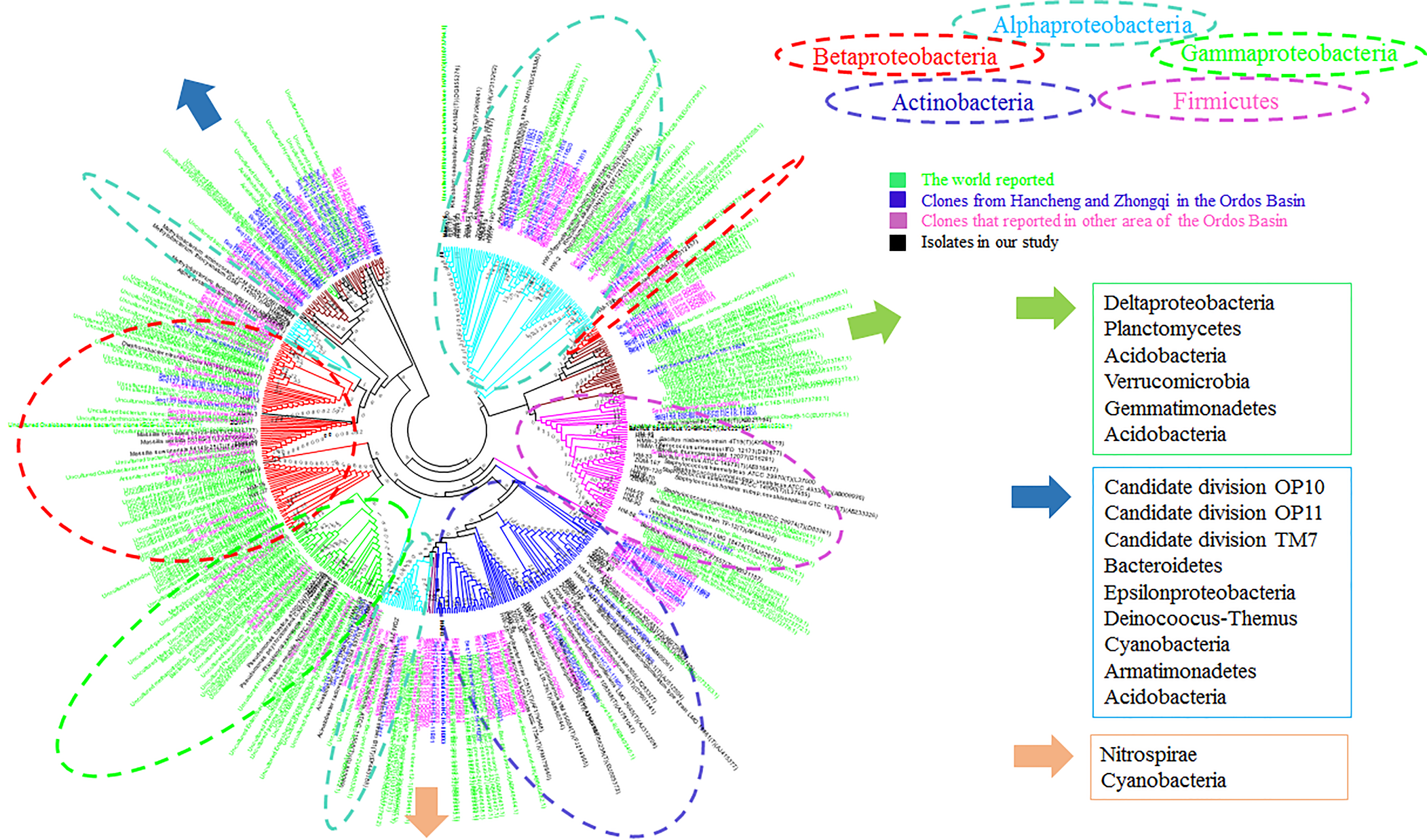
**

## References

Beckmann, S., Lueders, T., Kruger, M., Netzer, F.V., Engelen, B., Cypionka, H. (2011). Acetogens and acetoclastic *Methanosarcinales* govern methane formation in abandoned coal mines. Appl. Environ. Microbiol. 77 (11): 3749-3756. doi: 10.1128/AEM.02818-10

Doll, L., Moshitch, S., Frankel, G. (1993). Poly(GTG)_5_-associated profiles of *Salmonella* and *Shigella* genomic DNA. Res. Microbiol. 144 (1): 17-24. doi:10.1016/j.soilbio.2007.09.013

Kellner, H., Luis, P., Zimdars, B., Kiesel, B., Buscot, F. (2008). Diversity of bacterial laccase-like multicopper oxidase genes in forest and grassland cambisol soil samples. Soil Biol. Biochem. 40 (3), 638–648. doi:10.1016/j.soilbio.2007.09.013

Nicomrat, D., Dick, W.A., Tuovinen, O.H. (2006). Assessment of the microbial community in a constructed wetland that receives acid coal mine drainage. Microbial Ecol. 51 (1): 83-89. doi: 10.1007/s00248-005-0267-z

Ohtomo, Y., Ijiri, A., Ikegawa, Y., Tsutsumi, M., Imachi, H., Uramota, G.I., Hoshino, T., Morono, Y., Sakai, S., Saito, Y., Tanikawa, W., Hirose, T., Inagaki, F. (2013). Biological CO_2_ conversion to acetate in subsurface coal-sand formation using a high-pressure reactor system. Front. Microbiol. 4 (361): 1-16. doi: 10.3389/fmicb.2013.00361

Penner, T.J., Foght, J.M., Budwill, K. (2010). Microbial diversity of western Canadian subsurface coal beds and methanogenic coal enrichment cultures. Int. J. Coal Geol. 82 (1–2), 81–93. doi:10.1016/j.coal.2010.02.002

Shimizu, S., Akiyama, M., Naganuma, T., Fujioka, M., Nako, M., Ishijima, Y. (2007). Molecular characterization of microbial communities in deep coal seam groundwater of northern Japan. Geobiology 5 (4), 423–433. doi: 10.1111/j.1472-4669.2007.00123.x

Singh, D.N., and Tripathi, A.K. (2011). Evaluation of the Coal-Degrading ability of *Rhizobium* and *Chelatococcus* strains isolated from the formation water of an Indian Coal Bed. J. Microbiol. Biotechnol. 21 (11), 1101–1108. doi: 10.4014/jmb.1106.06005

Stepniewska, Z., Pytlak, A., Kuzniar, A. (2014). Distribution of the methanotrophic bacteria in the Western part of the Upper Silesian Coal Basin (Borynia-Zofiowka and Budryk coal mines). Int. J. Coal Geol. 130, 70–78. doi:10.1016/j.coal.2014.05.003

Versalovic, J.S.M., De Bruijn, F.J., Lupski, J.R. (1994). Genomic fingerprinting of bacteria using repetitive sequence-based polymerase chain reaction . Methods Mol. Cell Biol. 5, 25–40.

Wang, Y.N., Cai, H., Yu, S.L., Wang, Z.Y., Liu, J., Wu, X.L. (2007). *Halomonas gudaonensis* sp. nov., isolated from a saline soil contaminated by crude oil. Int. J. Syst. Evol. Microbiol. 57 (5), 911–915. doi: 10.1099/ijs.0.64826-0
